# The effect of the fat to starch ratio in young horses' diet on plasma metabolites, muscle endurance and fear responses

**DOI:** 10.1111/jpn.14037

**Published:** 2024-08-20

**Authors:** Saman Lashkari, Carina Beblein, Janne W. Christensen, Søren K. Jensen

**Affiliations:** ^1^ Department of Animal and Veterinary Sciences Aarhus University, AU Viborg, Research Centre Foulum Tjele Denmark; ^2^ Marketing Department Pavo Horse feed Vojens Denmark

**Keywords:** cortisol, glucose, insulin, lactate, α‐tocopherol

## Abstract

High‐starch diets may affect equine hindgut microbiota and increase blood glucose levels, which may cause unwanted physiological changes, but may also elicit behavioural changes such as increased fear reactions. The purpose of the current study was to feed a high starch (300) and low fat (43; HS_LF) or a low starch (60) and high fat (85; LS_HF, g/kg of DM) concentrate within the available commercial range and investigate how muscle endurance and fear reactions of horses respond to different diets. Twenty Danish Warmblood stallions (4 years) were randomly allocated to two treatments: LS_HF (*n* = 10) and HS_LF (*n* = 10) for 9 weeks. During the two last weeks, a single step exercise test was performed, and plasma metabolites and blood gases were measured before and after exercise in a 2 × 2 factorial design. The effect of two diets on fearfulness was tested by exposing the horses to novel objects test (T1 and T2). Plasma metabolites was not affected by diets. However, plasma level of glucose post‐exercise (4.9) was lower than pre‐exercise (5.6 mmol/L; *p* < 0.001). Similarly, plasma level of insulin post‐exercise (4.2) was lower than pre‐exercise (13.1 pmol/L; *p* < 0.001). Plasma level of lactate dehydrogenase (*p* < 0.001), non‐esterified fatty acids (*p* = 0.002), β‐hydroxybutyrate (*p* = 0.001), and fructosamine (*p* = 0.01) post‐exercise was higher than pre‐exercise. Regardless of type of diets, RRR‐α‐tocopherol was the dominance α‐tocopherol stereoisomers in plasma. In conclusion, during aerobic exercise, fat to starch ratio in horse diets within the normal range had no significant effect on plasma metabolites. However, horses fed LS_HF tended to show more investigative behaviour than horses fed HS_LF.

## 1 INTRODUCTION

Horses have evolved on scarce grasslands. The adaptation to these grasslands enables horses to generate energy from extraordinarily poor forage by bacterial fermentation in the hindgut (Kuntz et al., 2006). Today, most sport horses are only fed two to three times per day with an energy dense diet, to accommodate the energy demand from the muscles during exercise. Horses are often fed cereal based diets i.e. barley, wheat or maize (Vervuert et al., 2009), which are high in starch. Starch is broken down by α‐amylase and other digestive enzymes in the small intestine. If large amounts of starch are fed to horses, it may exceed the degradation capacity of digestive enzymes in the small intestine. This causes undigested starch to pass to the hindgut where it is rapidly fermented by bacteria. Fermentation of starch causes an imbalance between the volatile fatty acids, which lowers the pH in the hindgut (Geor et al., 2013). In addition, feeding horses with a 57% of hay and 43% of barley compared to a 100% hay diet changed the microbial activity, and resulted in changes in volatile fatty acids concentrations and increased lactate concentration in hindgut (Grimm et al., 2017). Grimm et al. (2017) reported a significantly higher lactate (both D and L forms) and lower pH in proximal hindgut in 57% of hay and 43% of barley diet compared to a 100% hay diet, respectively, which reflected the importance of volatile fatty profile. Low pH in the hindgut leads to negative effects on the horse's health such as overgrowth of undesired bacterial populations and lysis of desired bacterial populations and laminitis (Hoffman, 2009). Further, Vervuert et al. (2009) found that increasing amounts of starch, increased post‐prandial blood glucose and insulin levels. Hansen et al. (2020) reported that area under curve for glucose and insulin were higher in horses fed 2.77 compared to 0.07 g of starch/kg of body weight. Similar results have been found by Jansson and Lindberg (2012), who examined the effect of supply of only haylage or haylage and concentrate, and suggested that starch‐rich diets may promote insulin resistance in the horse, when exposed to high blood glucose levels for a long period of time. Insulin resistance is a potential risk factor associated with osteochondritis, Cushing's disease, colic and laminitis (Hoffman, 2009). Meyers et al. (1989) reported that adding fat to a diet could allow a reduction in feed intake, thereby reducing the risk of the above‐mentioned health issues (Delobel & Cuvelier, 2008). Similarly, Ropp et al. (2003) reported that increasing the fat level from 2.2% to 10.3% DM of diet on the expenses of non‐structural carbohydrate in the diet (decreasing form 44% to 24% DM of diet) resulted in lowered plasma glucose and insulin; however, such reduction in plasma glucose and insulin can be confounded effect between reduced intake of carbohydrate and effect of fat in the diet.

To evaluate physical performance, several studies have investigated the shift between aerobic and anaerobic metabolism and exercise at different intensities (Davie & Evans, 2000; Ronéus et al., 1994). The shift between aerobic and anaerobic metabolism, together with lactate accumulation in muscles, has been used as a measure of fitness in horses (Davie & Evans, 2000). Lactate accumulation is initiated during anaerobic metabolism and has been associated with muscle fatigue (Andrade & McMullen, 2006). To examine the shift between aerobic and anaerobic metabolism during exercise and when lactate accumulation occurs, blood lactate response to exercise at different intensities has been studied in horses (Andrade & McMullen, 2006; Ronéus et al., 1994). However, none of these studies had considered feed as a potential factor influencing the exercise capacity of the horse. Meyers et al. (1989) found that blood lactate levels tended to be lower, when fat was added to the horses' diets, and Jansson and Lindberg (2012) found that the absence of starch in the feed lowered blood lactate levels. Generally, the studies conducted examine more extreme amounts of starch, fat and training levels, than what typically is represented in practice. However, knowledge regarding how high and low starch and fat within the threshold of normal commercial feeds influences the horse during exercise is limited.

Behavioural reactivity has in some studies been connected to the type of diet (Hemmann et al., 2013). High‐starch diet can alter the equine hindgut microbiota, which may elicit behavioural changes via gut‐brain axis pathways (Bulmer et al., 2019). Lyte et al. (2016) in a mice study explained the gut‐brain axis pathways in which the resistance starch changed the behaviour of mice via altering the gut microbiota, and mice showed an increased anxiety‐like behaviour. Nicol et al. (2002) found that foals were more likely to develop crib‐biting when fed a high concentrate feed, and Freire et al. (2009) reported that horses fed a starch‐rich diet (50% oat) explored their stables less than horses fed a 35% grain diet, but explorative behaviour was not defined further. In addition, foals fed fat‐rich diets after weaning showed more investigative behaviour in a novel object test and were more likely to approach unknown people compared to foals fed starch‐rich diets (>35%) (Nicol et al., 2005). Redondo et al. (2009) reported that horses fed a fat‐rich diet were less startled by an umbrella opening compared to horses fed a starch‐rich diet (>40%). Finally, two studies by Bulmer and co‐workers showed that a starch‐rich diet led to increased heart‐rate responses in novel object and handling tests (Bulmer et al., 2015) and generally more nervous/reactive behaviour (Bulmer et al., 2019) compared to when the same horses were fed a low‐starch and high‐fibre diet in a cross‐over design. However, knowledge about how the fat and starch ratio typically found in commercial feeds affects muscle endurance and fear‐related behaviour is still limited. The objective of the present study was to evaluate the effect of fat to starch ratio on muscle endurance, fear‐related behaviour, and plasma metabolites of horses during aerobic exercise. It was hypothesised that horses fed a low starch/high fat diet would have better muscle endurance, show lower heart rate, differ in a range of plasma metabolites during exercise, and show reduced fear reactions.

## 2 MATERIALS AND METHODS

### 2.1 Animals and diets

Twenty, 4‐year‐old Danish Warmblood stallions were used in the present study. All horses were owned by and born at the same private stud and grew up under the same conditions. The horses had only been halter and lead trained for a previous study where all horses received the same amount of handling (Christensen et al., 2020).

The horses were housed in four group pens on straw bedding (*n* = 6 per pen) during winter without access to pasture. Twenty‐four horses were initially included into the study, but one had balance and leg coordination issues, which made the ramp on a treadmill an obstacle and a few showed dangerous behaviour during the initial habituation to the treadmill. Therefore, it was decided to use only five of the six horses per pen for the study. The study was carried out in a test arena (Figure S1) in a separate building that was connected to the stable. The treatments consisted of a high starch (300) and low fat (43; HS_LF) and a low starch (60) and high fat (85; LS_HF, g/kg of DM) concentrate. In HS_LF concentrate, starch was mainly originated by barely and oat, and in LS_HF, fat was supplied from vegetable sources (Table 1). The horses were randomly allocated to two treatment groups (LS_HF and HS_LF, i.e. treatment groups LS_HF in pen A1 and A2 and treatment groups HS_LF in pen B1 and B2; Figure S1) balanced according to sire and reactions in a baseline fear test. Of the 24 horses 21 was from the same sire and the remaining three from another sire. There was no significant difference in average body weight between the treatment groups (LS_HF: 522.2 ± 30.2 kg and HS_LF: 510.6 ± 55.2 kg, *p* = 0.58). The horses were assigned numbers together with a physical description, making identification possible. An additional pen with three horses was situated opposite the test arena; these horses were not included in the study but ensured that the test horses had visual contact to other horses during all training and testing. The pens alternated in treatments to minimise the effect of pen placement in the barn.

**Table 1:** Composition of feed ingredient of high starch/low fat (HS_LF) and low starch/high fat (LS_HF) concentrates (g/kg of DM).

	HS_LF	LS_HF
Barely	320.0	–
Lucerne	–	250.8
Oat	207.3	–
Wheat bran	120.0	91.2
Toasted full fat soybean	–	136.8
Corn germ	–	136.8
Dehulled sunflower meal	80.0	–
Grass pellets	60.0	–
Sugar beet pulp	60.0	205.2
Rapeseed meal	55.0	68.4
Sugar cane molasses	30.0	–
Fruit residues	–	79.8
Toasted soybean meal	25.3	–
Vegetable oil	15.0	–
Mineral and vitamin supplements	27.4	31.0

### 2.2 Feeding

**Figure 1:**
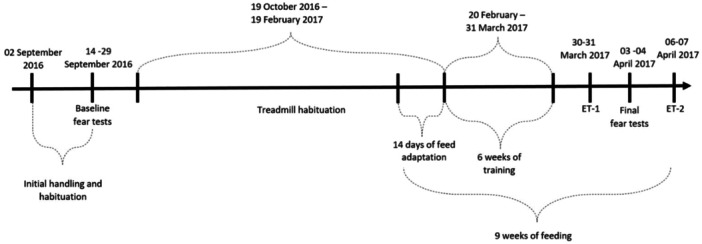
Overview of the time frame and structure of the study. Handling and habituation of the horses was carried out in September 2016. The baseline fear tests were conducted as soon as the horses met the habituation criterion for the test. The initial treadmill habituation started in October and the horses were ready for starting their 6 weeks of treadmill training by the end of February 2017. The 14 days of feed adaptation started on February 6th, ensuring that the horses had adapted to the feed for the start of the training period. After the treadmill training period, the final fear tests took place in the following week, whereafter two exercise tests took place. ET1 and ET2 were exercises 1 and 2, respectively, and results of exercise 2 were reported.

The horses were fed three kg of feed in both treatments daily and had free access to mature meadow hay of mixed composition, salt lick and automatic fresh water bowl. On training days, the horses were fed three times per day: in the morning, during training and in the evening. On days without training, the horses were fed twice a day (morning and afternoon). The horses were fed individually in buckets at least once a day to ensure that all horses at least consumed 1.5 kg of feed. The other feeding was in a group feeding trough; it was ensured; however that all horses were allowed to eat. An overview of the time frame and structure of the study is shown in Figure 1.

### 2.3 Physical performance

Effect of the two concentrate (LS_HF and HS_LF) on the horses’ performance on a treadmill (Hoffmann, Oldenburger) was tested. Prior to initiating the treadmill training, the horses were habituated to enter and walk on the treadmill. The habituation to the treadmill was divided into 11 stages (Table S2), and after completing the 11 stages, the horse was considered ready for the treadmill training. Treadmill habituation training took place 2–3 days/week for 4 months. The horses met the criteria for the habituation at different times. Therefore, the horses which had met the criteria for the habituation to the treadmill, maintained the habituation training once a week until testing.

The horses had a total of 22 treadmill training days distributed four times a week for 5 weeks and two times in the sixth week. Treadmill training was divided into six sub programs (Table S2), which was adjusted to the fact that the horses had not received any previous physical training. During training, the handler had a bag around the waist with one kg of feed, which was offered to the horses during exercising in order to keep the motivation for entering the treadmill. During a training session, most horses ate half a kg of the feed assigned to them (LS_HF or HS_LF). They ate the remaining feed after finished exercise. The exercise test was performed over 2 consecutive days with 12 horses on each day. Before initiating the exercise test, horses were equipped with heart rate monitoring equipment (Polar Equine RS800CX G3), measuring the heart rate during the exercise on the treadmill. The heart rate monitoring succeeded on 10 horses per day. Blood samples were taken from Jugular vein immediately before the horse entered the treadmill and immediately after it left the treadmill. A separate whole blood sample was immediately analysed for blood gasses (O_2_ and CO_2_), and the rest of blood sample was stored on ice and centrifuged at 3500 rpm/min (equal to 2058 g) at 4°C for 15 min, and plasma was collected and stored at −20°C for further plasma analysis.

### 2.4 Behaviour

During the experiment, the horses were minimally handled and had been handled only for necessary veterinary or farrier treatment since taking part in a behavioural study as yearlings. Initial handling and habituation were, therefore, necessary before the baseline fear tests could be conducted. The habituation to handling was divided into five stages (Table S3). When a horse had completed the five stages, it was considered ready for the baseline fear tests; i.e. when released in front of the test arena it walked directly to the feed container placed opposite the entrance and started to eat.

### 2.5 Baseline fear tests

Prior to the onset of the feeding treatments, the horses were tested for their level of fearfulness in two novel object tests (Figure 1). The methodology and results from the baseline fear tests are published in Christensen et al. (2020) and were used to balance the horses on the two treatments, ensuring that the treatment groups did not differ in reactivity prior to the onset the feeding treatment (LS_HF vs. HS_LF: *p* > 0.05 for all behavioural and heart rate variables).

### 2.6 Final fear tests

In the final week of the feeding treatments (Figure 1), the horses were again tested for their level of fearfulness in two novel object tests. Before testing, the horses were habituated to entering the test arena on their own when released in the doorway by the handler. A feed container with the horses’ usual food was placed opposite the entrance and a horse was considered ready for testing when it walked directly to the feed container and ate for 1 min. In the first object test, the horses were exposed to two open umbrellas placed 0.5 m from the feed container. In the second object test, white and shiny plastic (1.4 × 3 m) was placed on the floor between the entrance and the feed container, and four boxes (two pink and two blue [width; height; length: 0.4 m; 0.4 m; 0.6 m]) were placed on the corners. There was a 1 m gap between the objects and the arena wall on each side, i.e. the horse could walk either around or across the plastic to approach the feed container. Before the start of the first object test, the horse was equipped with heart rate monitoring equipment (Polar Equine RS800CX G3) in the stable corridor. The test time (120 s) started when the horse was released in the doorway. After T1, the horse was led back into the corridor and the object was removed from the arena. The horse was then led back to the arena and allowed to eat for approximate 10 s before returning to the corridor. The test arena was subsequently equipped with objects for T2. Again, the test time (120 s) started when the horse was released in the doorway. Both tests were recorded on video for later behavioural analysis (Table S4).

### 2.7 Analysis of feed and plasma metabolites

The concentrate and hay were analysed for chemical composition. Dry matter content was measured by oven drying at 60°C for 48 h. Nitrogen was determined by the Dumas method (Hansen, 1989) and crude protein was expressed as nitrogen multiplied by 6.25. After HCl hydrolysis of samples, crude fat was determined by petroleum ether extraction (Stoldt, 1952) using a Soxtec system. Starch was analysed according to the acetate buffer method (Hall, 2009), with the modifications that samples were incubated at 50°C instead of 60°C, 3.0 mL of glucose oxidase‐peroxidase reagent was used instead of 2.5 mL, and the last incubation was done at 50°C for 20 min. Neutral detergent fibre, acid detergent fibre and acid detergent lignin were determined by using α‐amylase and sodium sulfite on a Fibertech system and corrected for residual ash (Mertens, 2002). Phosphorous and calcium concentrations of feedstuffs were determined after acidification in nitric acid and destruction at 1500 W at 230°C for 25 min, in a microwave digestion system (Ultra Wave, single reaction chamber, Milestone). Low molecular weight sugars were measured using a modification of the method described by Knudsen and Li (1991). Briefly, 10–15 mL of 50% ethanol (vol/vol) containing 1 mg/mL of arabinose as internal standard were added to 500 mg of feed samples, and the samples were sonicated and extracted in a water bath for 60 min at 65°C. Then samples were mixed (vortex mixer) and finally centrifuged (2200*g*, 20 min). A 5 mL of supernatant was taken and filtered through a Bond Elude C18 cartridge (Analytichem International) pre‐wetted with methanol (2 mL) and deionized water (5 mL). Afterwards, the first 1.5 mL of eluate was collected and filtered through a 0.22 1‐Lm PTFE filter (Minisart NMLPF, Sartorius AG), taken to dryness under vacuum at 50°C and 20 µL used for HPLC analysis.

Plasma concentration of glucose, lactate, triglycerides, albumin, total protein, alanine aminotransferase, aspartate aminotransferase, creatine kinase, and alkaline phophatase was determined according to standard procedures (Siemens Diagnostics Clinical Methods for ADVIA 1650), and non‐esterified fatty acids according to the Wako, non‐esterified fatty acid C ASA‐ACOD assay method (Wako Chemicals GmbH) using an auto analyser (ADVIA 1650 Chemistry System; Siemens Medical Solutions). Plasma β‐hydroxybutyrate was determined as an increase in absorbance at 340 nm due to the production of NADH at slightly alkaline pH in the presence of β‐hydroxybutyrate dehydrogenase, and a blank sample was included. Insulin was determined by time‐resolved fluoro‐immunometric assay as described by Løvendahl and Purup (2002). Plasma cortisol was determined by EIA, K003‐H1, Arbor Assays. Insulin‐like growth factor 1 was determined on acid/ethanol extracted serum, including a cryoprecipitation step (Juul et al., 1994). Plasma calcium was analysed according to standard procedures (Siemens Diagnostics R Clinical Methods), using an auto‐analyser, ADVIA 1800 R Chemistry System (Siemens Medical Solutions). Plasma fructosamine was determined by a colorimetric assay (reduction of nitrotetrazolium‐blue), Roche Diagnostics GmbH, D‐68298 Mannheim. Blood samples were analysed for blood gases by using RapidPoint 500 System Gas Analyzers (Siemens Healthcare Diagnostics) immediately after sampling.

The concentration of α‐tocopherol and α‐tocopherol stereoisomers in individual feedstuffs and plasma was analysed after saponification and extraction into heptane by HPLC according to Jensen et al. (1999). A PerkinElmer HS‐5‐Silica column (4.0 × 125 mm) was used for α‐tocopherol analyses. Separation of stereoisomers of α‐tocopherol was performed on a Chiralcel OD‐H column (250 × 0.46 mm, 5 µm particle size), cellulose tris (3,5‐dimethylphenylcarbamate) from Daicel Chemical industries with heptane as eluent. This method allowed to separate of the eight stereoisomers of α‐tocopherol into five peaks of which the first peak was the SRR, SSR, SRS and SSS stereoisomers; the second peak was the RSS; the third peak was RRS; the fourth peak was RRR and the fifth peak containing RSR.

### 2.8 Statistical analysis

Statistical analysis of plasma metabolites and blood gases were performed in SAS software (version 9.1, release 8.02, SAS Institute). The statistical model used was a general linear model as follows:

$$Y_{\mathrm{ijk}}=µ+\alpha_{i}+\beta_{j}+{\alpha \beta }_{\mathrm{ij}}+\upsilon_{k}+\varepsilon_{\mathrm{ijk}},$$

where *Y* is dependent variable, *μ* is the overall mean, and the model includes the fixed effects of diets (*α*_*i*_; HS_LF and LS_HF), exercise (*βj*; per‐exercise and post‐exercise), the interaction between diet and exercise (*αβ*_*ij*_), random effect of animal $\left(\upsilon_{k}\right)$, and ε_ijk_ was the residual error. These random effects are assumed to be independent and normal distributed with zero mean and variances $\sigma_{p}^{2}$, $\sigma_{r}^{2}$, and $\sigma_{\varepsilon }^{2}$ respectively. Significance was chosen at *p* < 0.05 and tendencies at 0.05 ≤ *p* ≤ 0.10. When interaction between diet and exercise was significant, least squares means were compared using the Tukey‐Kramer test at *α* = 0.05. All results presented are Ls‐means ± standard error unless otherwise noted (Tables 1 and 2). In addition, all the presented results in the results section are Ls‐means ± standard error of the main effects.

**Table 2:** Nutrient composition of high starch/low fat (HS_LF) concentrate, low starch/high fat (LS_HF) concentrate, and hay (g/kg of DM unless otherwise noted; means ± SD of duplicate).

	HS_LF	LS_HF	Hay
Dry matter (%)	914 ± 0.8	923 ± 6.9	921 ± 2.1
Crude protein	166 ± 0.0	137 ± 1.3	124 ± 1.8
Neutural detergent fibre	266 ± 4	310 ± 1	711 ± 0.1
Acid detergent fibre	136 ± 0.8	201 ± 2	388 ± 2.1
Acid detergent lignin	29 ± 4	48 ± 2	56 ± 1.1
Starch^a^	305	51	3.6
Crude fat^a^	43	85	NA^b^
Fructose^a^	2.5	8.1	0.63
Fructan^a^	6.3	2.3	0.35
Glucose^a^	2.5	5.2	0.45
Sucrose^a^	39	93	0.1
Total sugar^a^	51	108	15
Calcium	13 ± 0.5	23 ± 0.3	5.6 ± 0.03
Phosphorous	6.7 ± 0.03	5.8 ± 0.08	3.32 ± 0.02
α‐Tocopherol (mg/kg)	307 ± 0.1	291 ± 5.7	98 ± 6
Composition of α‐tocopherol stereoisomers (%)^a^			
RRR	13.3	16.6	100.0
RRS	12.9	13.1	0
RSS	12.4	11.6	0
RSR	12.2	11.5	0
2S	49.2	47.2	0

^a^ One sample was analysed.

^b^ Not analysed.

Plasma alanine aminotransferase, aspartate aminotransferase, and α‐tocopherol as percentage of triglyceride were not normally distributed. Plasma alanine aminotransferase was transformed by 1 over squared, and plasma aspartate aminotransferase and plasma α‐tocopherol as percentage of triglyceride were transformed by log_e_. However, the back transform data and confidence interval [25;75% quartiles] were reported in the text or tables.

For the fear tests, the response variables (average of heart beats, maximum heart beats, latency to feed, object focus, object touch and sniffing), and maximum heart rate, average heart rate during exercise were analysed for an effect of treatment (LS_HF vs. HS_LF) in a *t*‐test. The video analysis and reading of heart rate data were performed by a person who was blind regarding the treatments. If data did not meet the assumptions for the model (normal distribution and variance homogeneity), the Mann–Whitney test was used. Normal distribution was assessed from plots and the Shapiro–Wilks test. Data is presented as; “least square means ± SEM” or “median [25;75% quartiles]” for data that did not meet the normal distribution criteria. A *p* < 0.05 was considered significant and *p* < 0.10 was considered as tendency. Data were analysed in SigmaPlot13 (www.systat.com).

## 3 RESULTS

### 3.1 Diet composition

Composition of the two concentrates and hay is shown in Table 2. Starch content in HS_LF and LS_HF is in line with the planned starch content. Starch content was increased from 51 in LS_HF to 300 g/kg of DM in HS_LF. Correspondingly, the crude fat level was increased from 43 in HS_LF to 85 g/kg of DM in LS_HF. Calcium content of LS_HF was higher than HS_LF. The α‐tocopherol content was 307 and 291 mg/kg of feed in HS_LF and LS_HF, respectively, and the α‐tocopherol stereoisomer composition showed that the synthetic α‐tocopherol was included as a source of α‐tocopherol in commercial horse diets. The hay was characterised by a high fibre content and a low content of sugars.

### 3.2 Plasma metabolites and blood gases

Plasma metabolites of horses fed different types of diets at pre‐ and post‐exercise are presented in Table 3. Plasma glucose level was not affected by diets. However, plasma glucose level was affected by exercise (*p* < 0.001) and it was higher pre‐exercise (5.6 ± 0.08; Ls‐means ± SEM) than post‐exercise (4.9 ± 0.08 mmol/L). Plasma lactate was not affected by diet and exercise without interaction between diet and exercise. Plasma lactate dehydrogenase level was affected by exercise without interaction between diet and exercise and it was higher (385 ± 18) post‐exercise than pre‐exercise (368 ± 18 U/L).

**Table 3:** Plasma metabolites and blood gases of horses fed high starch/low fat (HS_LF) or low starch/high fat (LS_HF) concentrates at pre‐ and post‐exercise.

		HS_LF		LS_HF			*p* values		
	Baseline^a^	Pre‐exercise	Post‐exercise	Pre‐exercise	Post‐exercise	SEM or CI^b^	Diet	Exercise	Diet × exercise
Glucose (mmol/L)	5.95 ± 0.13	5.55	4.94	5.35	4.84	0.12	0.26	<0.001	0.67
Lactate (mmol/L)	0.79 ± 0.24	0.85	0.85	0.78	0.84	0.06	0.80	0.60	0.08
Lactate dehydrogenase (U/L)	315 ± 12	371	388	364	381	26	0.84	<0.001	0.95
Triglyceride (mmol/L)	0.23 ± 0.01	0.27	0.29	0.31	0.33	0.02	0.14	0.01	0.63
Non‐esterified fatty acids (µekv/L)	36.2 ± 11.3	20.8	43.4	22.9	69.3	10.7	0.24	0.002	0.25
β‐hydroxybutyrate (mmol/L)	0.23 ± 0.01	0.26	0.24	0.28	0.25	0.02	0.18	0.001	0.43
Insulin (pmol/L)	14.5 ± 1.5	15.2	4.2	10.9	4.1	2.5	0.47	<0.001	0.27
Fructosamine (µmol/m)	286 ± 4	282	287	275	288	5	0.65	0.01	0.26
Insulin‐like growth factor 1 (ng/mL)	146 ± 11	152	177	169	155	19	0.90	0.75	0.30
Cortisol (µg/L)	28.2 ± 2.4	29.2^b^	29.1^b^	32.8^b^	45.5^a^	5.4	0.18	0.007	0.006
Total protein (g/L)	68.9 ± 1.0	70.5	72.4	68.2	71.4	1.1	0.26	<0.001	0.23
Albumin (g/L)	35.4 ± 0.5	34.8	35.9	34.5	36.2	0.5	0.94	<0.001	0.29
Calcium (mmol/L)	3.02 ± 0.03	3.11	3.08	3.24	3.17	0.04	0.04	0.04	0.65
Creatine kinase (U/L)	253 ± 21	235	258	299	301	24	0.08	0.47	0.56
Alkaline phosphatase (U/L)	163 ± 6	153	155	160	168	8.0	0.36	0.03	0.24
Alanine aminotransferase (U/L)^c^	9.0 ± 0.5	11.2	12.0	11.3	10.9	10.2–12.7	0.58	0.43	0.74
Aspartate aminotransferase (U/L)^c^	285 ± 6	303	313	299	315	293–355	0.91	0.18	0.73
O_2_ (%)	NA^d^	30	33	30	35	1	0.35	0.003	0.44
CO_2_ (%)	NA^d^	42.6	42.3	43.7	42.7	3.0	0.97	0.26	0.37
α‐Tocopherol (% of triglyceride)^c^	8.5 ± 0.8	5.2	4.9	4.3	4.3	3.2–6.0	0.30	0.87	0.83
α‐Tocopherol (µg/mL)	1.8 ± 0.1	1.4	1.6	1.4	1.5	0.2	0.83	0.007	0.36
α‐Tocopherol stereoisomers (% of total α‐tocopherol)									
RRR	67.9 ± 2.3	61.2	58.9	71.4	72.8	2.3	0.001	0.70	0.11
RRS	9.7 ± 0.6	13.9	14.7	10.4	10.1	0.8	0.001	0.35	0.05
RSS	7.6 ± 0.5	10.0	10.9	7.7	6.9	0.7	0.003	0.94	0.07
RSR	7.4 ± 0.5	9.62	9.48	7.2	7.1	0.6	0.005	0.59	0.98
2S	7.4 ± 0.6	5.3	6.0	3.2	3.1	0.4	0.006	0.57	0.39

Abbreviation: CI, confidence interval.

^a^ Baseline is the values in the beginning of the experiment before feeding the HS_LF and LS_HF (Means ± standard error of means).

^b^ Standard error of means or confidence interval [25;75% quartiles].

^c^ Not normally distributed.

^d^ Not analysed.

Means in the same row with different superscripts differ (*p* ≤ 0.05).

Plasma triglyceride level post‐exercise (0.31 ± 0.01) was higher than pre‐exercise (0.29 ± 0.01 mmol/L; *p* = 0.01). Plasma non‐esterified fatty acid level was not influenced by diet while non‐esterified fatty acid level post‐exercise (57 ± 8) was higher than pre‐exercise (22 ± 7 mmol/L; *p* = 0.002). Similarly, plasma β‐hydroxybutyrate level was unaffected by diet while plasma β‐hydroxybutyrate level post‐exercise (0.27 ± 0.01) was lower than pre‐exercise (0.24 ± 0.01 mmol/L; *p* = 0.001). Plasma insulin level was unaffected by diets; however, plasma insulin level post‐exercise (4.2 ± 1.7) was lower than pre‐exercise (13.1 ± 1.7 pmol/L; *p* < 0.0001).

Even though the plasma fructosamin level was not affected by diet, plasma fructosamin level post‐exercise (287 ± 4) was higher than pre‐exercise (279 ± 4 µmol/L; *p* = 0.01) without interaction between diet and exercise. For plasma cortisol level, there was an interaction between diet and exercise (*p* = 0.006) and it was higher in LS_HF post‐exercise than the LS_HF pre‐exercise, HS_LF pre‐exercise, and HS_LF post‐exercise. Plasma total protein level was affected by exercise (*p* < 0.001) and it was higher post‐exercise (72 ± 0.8) than pre‐exercise (69 ± 0.8 g/L). Similar to plasma total protein, plasma albumin level was higher post‐exercise (36 ± 0.3) than pre‐exercise (34 ± 0.3 g/L; *p* < 0.001). Plasma calcium level was affected by diet (*p* = 0.04) and exercise (*p* = 0.04). Plasma calcium level in LS_HF (3.2 ± 0.4) was higher than HS_LF (3.1 ± 0.03 mmol/L). In addition, plasma calcium level in pre‐exercise (3.2 ± 0.03) was higher than post‐exercise (3.1 ± 0.03 mmol/L). Plasma creatine kinase level in LS_HF (300 ± 20) tended to be higher than HS_LF (262 ± 20 U/L; *p* = 0.08). Plasma alkaline phosphatase level was affected by exercise (*p* = 0.03) and it was higher post‐exercise (162 ± 6) than pre‐exercise (157 ± 6 U/L). Plasma level of insulin‐like growth factor 1, alanine aminotransferase and aspartate aminotransferase was not affected by either diet or exercise. Plasma O_2_ level post exercise (34 ± 0.9) was higher than pre‐exercise (30 ± 0.9%; *p* = 0.003). However, plasma CO_2_ was not affected by either diet or exercise.

Average heart rate during the exercise was 109 ± 3 and 121 ± 8 beats per minute (*p* = 0.25; results not presented in tables) in LS_HF and HS_LF respectively. Likewise, maximum heart rate was similar (*p* = 0.75) between LS_HF and HS_LF (140 ± 4 and 143 ± 7 beats per minute, respectively; results not presented in tables).

### 3.3 Plasma α‐tocopherol level and stereoisomer composition

Plasma α‐tocopherol expressed as a proportion of plasma triglycerides was not affected by diet or exercise. However, plasma α‐tocopherol post‐exercise (1.5 ± 0.1) was higher than pre‐exercise (1.4 ± 0.1 µg/mL; *p* = 0.007). Plasma proportion of RRR‐α‐tocopherol in LS_HF (72 ± 2) was higher than HS_LF (66 ± 2%; *p* = 0.001). For RRS and RSS‐α‐tocopherol, there was a tendency for interaction between diets and exercise (*p* = 0.05 and 0.07, respectively). Regardless of diets, the RRR‐α‐tocopherol stereoisomer occurred in the highest proportion in plasma followed by RRS, RSS, RSR, and 2S‐α‐tocopherol.

### 3.4 Behaviour

No significant differences were found between the treatment groups in the fear tests. However, in the second object test, horses fed LS_HF tended to have a longer latency to eat and to spend more time sniffing the objects compared to horses fed diet HS_LF (Table 4).

**Table 4:** Behavioural and heart rate reactions of horses fed a low starch/high fat diet (LS_HF) or a high starch/low fat diet (HS_LF) concentrates in two object tests.

	Object test 1 (T1)^a^				Object test 2 (T2)^a^			
	LS_HF	HS_LF	SEM or CI^b^	*p* value	LS_HF	HS_LF	SEM or CI^b^	*p* value
Average heart rate (beats/min)	60.1	59.0	4.6	0.84	53.8	52.8	2.5	0.76
Maximum heart rate (beats/min)	90.3	99.7	8.1	0.36	82.5	84.4	7.0	0.83
Latency (s)^c^	17.0	23.0	12.8–82.5	0.99	30.5	16.0	19.8–45.8	0.09
Object focus (s)^c^	13.5	17.0	6.3–37.8	0.79	8.0	3.5	2.0–18.0	0.38
Touch (s)^c^	0.0	0.0	0–0.5	0.17	0.0	0.0	0–2.5	0.93
Sniff (s)^c^	5.5	3.5	2.8–7.0	0.65	2.0	0.0	0.8–2.3	0.07

Abbreviation: CI, confidence interval.

^a^ Object test 1 (T1) was two open umbrellas placed 0.5 m from the feed container, and object test 2 (T2) was white, shiny plastic (1 × 4 m) placed on the floor between the entrance and the feed container and four boxes (two pink and two blue [width; height; length 0.3 m; 0.3 m; 0.4 m]) placed on the corners.

^b^ Standard error of means or confidence interval [25;75% quartiles].

^c^ Not normally distributed.

## 4 DISCUSSION

Several studies generally recommended that regardless of starch source, horses should not be fed more than 2 g/kg body weight of starch per meal, corresponding to 1 kg of starch for a 500 kg horse, due to the risk of undigested starch reaching the hindgut (Geor et al., 2013; Vervuert et al., 2009). Further, it has been shown that 20% of the ingested starch reached the hindgut of horses fed more than 1 g starch/kg body weight per meal (Geor et al., 2013). Luthersson et al. (2009) demonstrated that horse should be fed less than 1 g/kg of body weight per meal to prevent equine gastric ulceration syndrome and nonglandular ulcers. In the present study, horses was fed 3 kg of concentrate/day, containing 300 g of starch/kg dry matter of concentrate, mainly from barley and oat, corresponding to 0.59 g of starch intake/kg of body weight per meal during the blood/plasma sampling. Thus, the HS_LF did not exceed the starch intake and it is not likely that undigested starch reached the horses’ hindgut. In the present study, horses had free access to hay containing 3.6 g starch/kg of DM. According to National Research Council (NRC, 2007), grass hay intake is around 2.0–2.1 kg/100 kg of BW. However, it was estimated that the horses consumed 7–9 kg of hay per day, providing around 30 g of starch intake in HS_LF, which was negligible amount compared to 900 g per day from HS_LF concentrate. Further, the levels of fat did not exceed the 15% of total dry matter of diet recommended by National Research Council (NRC, 2007) to minimise the risk of reduced fibre digestibility due to fat overload. Ratio of fat and starch in the present study reflects the variation in commercially available horse feed.

### 4.1 Plasma metabolites

Significantly lower plasma glucose level post‐exercise compared to pre‐exercise shows the use of glucose for muscle contraction. In contrast to our findings, Gordon et al. (2007) reported an increased plasma glucose level immediately post‐exercise. The discrepancy between our results and Gordon et al. (2007) could be due to the type of exercise. Gordon et al. (2007) exercised the horses on a high‐speed treadmill, where the intensity was increased until fatigue activating hepatic gluconeogenesis, while in the present study the horses were not pushed until fatigue. In addition, nonsignificant effect of diet on plasma glucose level could be explained by a relatively short treatment period. Zeyner et al. (2002) had a long treatment period (390 days) and found higher plasma glucose level in horses fed a starch‐rich diet compared to horses fed a low starch diet. Higher plasma insulin level pre‐exercise than post‐exercise shows the significance of insulin to regulate blood glucose level.

Lack of exercise effect on plasma lactate and creatine kinase level reflects that the level of activity in horses was lower than fatigue. Nonsignificant exercise effect on plasma lactate level is in line with increased plasma lactate dehydrogenase level, which may reflect the activity of lactate dehydrogenase to convert lactate generated from exercise to pyruvate. Plasma lactate level indicated that the energy requirement during exercise was ensured by aerobic metabolism, because the values in general were below 2 mmol/L (Vincze et al., 2016). During aerobic exercise, both starch and fat can be utilised, whereas under anaerobic conditions, fat is not a possible energy source (Lindner et al., 2009). Thus, in the current study, it was important to keep the horses on aerobic metabolism to promote aerobic fatty acid oxidation. In addition, higher plasma non‐esterified fatty acid level accompanied by lower plasma β‐hydroxybutyrate level in post‐exercise compared to pre‐exercise likely reflect that the supply of energy requirement occurred in the aerobic stage. Higher plasma non‐esterified fatty acid level post‐exercise compared to pre‐exercise clearly showed that fatty acids had a remarkable role as energy supplier during the exercise. In addition, the 47% increase in plasma non‐esterified fatty acid level in LS_HF compared to HS_LF demonstrated considerable higher energy supply from fatty acids during the exercise. The highest cortisol level in LS_HF post‐exercise could be related to energy supply from oxidation of poly‐unsaturated fatty acids during the exercise, which may increase thiobarbituric acid reactive substances as indicator to oxidative stress of tissues (Granit et al., 2001). A positive and significant correlation has been reported between thiobarbituric acid reactive substances and plasma cortisol (Wernicki et al., 2006).

Plasma calcium level in LS_HF was higher than HS_LF, which could be due higher calcium content in LS_HF, despite the risk for formation of calcium soap of fatty acids due to both higher calcium and fatty acids in LS_HF compared to HS_LF. Calcium has an important role regarding fatigue during exercise. In the present study, the level of exercise did not press the horses to the level of fatigue, which can be an explanation for the lower plasma calcium level in post‐exercise than pre‐exercise.

The higher post‐exercise plasma albumin, total protein, and fructosamine than pre‐exercise can be explained by plasma water loss during exercise (Aguilera‐Tejero et al., 2000). Increased plasma albumin level in post‐exercise is in line with increased plasma non‐esterified fatty acid level, because albumin is a plasma carrier protein for non‐esterified fatty acid (Aguilera‐Tejero et al., 2000). In addition, increased plasma fructosamine level post‐exercise is also in line with increased albumin level in LS_HF in post‐exercise compared to pre‐exercise, because fructosamine is a glycosylated plasma protein, primarily albumin (Reusch et al., 1993).

Neither plasma alanine aminotransferase nor aspartate aminotransferase was affected by diet or exercise. It has been reported that increased level of these two plasma enzymes is due to increase permeability of both enzymes from muscle cells due to muscular stress (Watson, 1998). However, the relatively low intensity of exercise in the present experiment can explain the lack of exercise effect on plasma alanine aminotransferase and aspartate aminotransferase. High intensity training causes increased aspartate aminotransferase within 5 min after exercise, as shown in thoroughbred race horses (Allaam et al., 2014).

### 4.2 Plasma α‐tocopherol level and stereoisomer composition

It has been demonstrated that effort situations such as exercise cause increased free radicals and lipid peroxidation, which results in an increased antioxidant demand in stressed cells (Mills et al., 1996). In the present study, the plasma α‐tocopherol measured in horses was not influenced by the exercise intensity. It was expected that even a small pressure on the antioxidant demand would have reduced the level of α‐tocopherol (Avellini et al., 1995). In agreement with our results, no significant difference was observed in red blood cell glutathione peroxidase and superoxide dismutase level before compared to after competing in two 1‐min runs of intense exercise over jumps (Balogh et al., 2001); however, Balogh et al. (2001) did not measure plasma α‐tocopherol. Effect of exercise on α‐tocopherol level might have been more evident if we could measure the α‐tocopherol at muscle level instead of plasma level. Another explanation for nonsignificant effect of exercise on plasma α‐tocopherol can be due to sparing effect of other antioxidant compounds such as vitamin C or glutathione peroxidase on α‐tocopherol.

Regardless of diets, composition of plasma α‐tocopherol stereoisomers demonstrated an obvious dominance of 2R stereoisomers in the following order: RRR > RRS > RSS > RSR > 2S due to affinity of α‐tocopherol transfer protein toward the 2R configuration. This finding is in agreement with findings from other species as human (Blatt et al., 2004), cow (Jensen et al., 2020), calf (Lashkari et al., 2021b, 2022), lamb (Lashkari et al., 2019), and mink (Hymøller et al., 2018; Lashkari et al., 2021a). In these species administration of synthetic form of α‐tocopherol shows discrimination against the 2S‐α‐tocopherol, maintaining the highest proportion of 2R stereoisomers in plasma due to elimination of 2S‐α‐tocopherol stereoisomers in the liver (Leonard et al., 2002; Traber et al., 1990), resulting in plasma 2R stereoisomers enrichment.

### 4.3 Behaviour

It was hypothesised that horses fed HS_LF would show stronger fear reactions, compared to horses fed LS_HF, when a novel object was presented. Stronger fear reactions would be reflected in increased heart rates, a longer latency to eat from the feed container and increased object focus. However, no significant differences were found in neither behavioural nor physiological responses in the fear tests. Thus, we failed to replicate previous results where horses were shown to be less reactive when fed a starch‐reduced diet (Bulmer et al., 2015, 2019). The discrepancy between our finding could be explained by the higher starch level (0.7 g of starch/kg BW per meal) in the study by Bulmer et al. (2015) and the higher starch level (0.96 g of starch/kg BW per meal) in the study by Bulmer et al. (2019) than the starch level in our study. In addition, the discrepancies can be caused by several reasons, including experimental design with Bulmer and co‐workers applying a cross‐over design; differences in the intensity of the fear tests, and breed of the horse. However, in our study horses fed LS_HF tended to show a longer duration of sniffing the objects and a corresponding longer latency to eat from the feed container in T2. Increased sniffing may be indicative of curiosity towards the objects, which in turn can lead to an increased latency to eat. This is in line with previous studies by Freire et al. (2009) where horses fed a low‐starch diet showed more exploratory behaviour.

## 5 CONCLUSION

The results of the present study showed an interaction between exercise and diet for plasma cortisol level, and LS_HF had the highest plasma cortisol. Plasma creatine kinase tended to be higher in LS_HF than HS_LF. However, during aerobic exercise, fat to starch ratio in horse diets within the commercially available range had no remarkable effect on other measured plasma metabolites. The results demonstrated lowered plasma insulin and glucose levels post‐exercise and increased plasma non‐esterified fatty acids, β‐hydroxybutyrate, and triglycerides. In addition, no differences in fear reactions were found between the diets; however, horses fed LS_HF tended to show more investigative behaviour than horses fed HS_LF.

## AUTHOR CONTRIBUTIONS

**Saman Lashkari**: Data curation, formal analysis, investigation, methodology, software, validation, writing—original draft, writing—review and editing. **Carina Beblein**: Investigation, methodology, resources, validation, visualisation, writing—original draft, writing—review and editing. **Janne W. Christensen**: Data curation, formal analysis, investigation, methodology, software, methodology, validation, writing—review and editing, writing—original draft. **Søren K. Jensen**: Investigation, data curation, methodology, resources, validation, visualisation, writing—original draft, project administration, writing—review and editing.

## CONFLICT OF INTEREST STATEMENT

The authors declare no conflicts of interest.

## Supporting information

Supporting information.

## Data Availability

Data will be provided through direct contact with corresponding authors. The data that support the findings of this study are available from the corresponding author upon. reasonable request.
