# Supplementary material for: The effect of the fat to starch ratio in young horses' diet on plasma metabolites, muscle endurance and fear responses
Source: J Anim Physiol Anim Nutr (Berl). 2024 Aug 20;109(1):113–23. doi: 10.1111/jpn.14037 (PMC11731427; doi:10.1111/jpn.14037)
Supplement: Supplementary file 1 — Supporting information. [file JPN-109-113-s001.docx]

Figure S1. Overview of the stable and experimental area. The grey area is where all training and tests (object tests and treadmill training) were carried out. The yellow bar demonstrates the entrance and exit of the test arena. The corridor was used for the initial handling of horses as well as for fitting of heart rate equipment etc. for the fear tests. Pens A and B contain the horses for this study (n5/pen). Horses in the last ‘pen’ were not used for this study.

Table S2. The training programs used during the 6 weeks of training.

| Program | Training intensity |
| --- | --- |
| 1 | • 2.5 minutes warm up on the treadmill at speed 4.8 km/h (level 0)  • 5 minutes on the treadmill at speed 5.2 km/h (level 1)  • 5 minutes on the treadmill at speed 6.2 km/h (level 2)  • 2.5 minutes of cooling down on the treadmill 4.8 km/h (level 0)  Total time: 15 min |
| 2 | • 4 minutes of warm-up on the treadmill at speed 5.2 km/h (level 1)  • 5 minutes on the treadmill at speed 6.2 km/h (level 2)  • 5 minutes on the treadmill at speed 7.8 km/h (level 3)  • 4 minutes of cooling down on the treadmill 5.2 (level 1)  Total time: 18 min |
| 3 | • 4 minutes of warm-up on the treadmill at speed 5.2 km/h (level 1)  • 4 minutes on the treadmill at speed 6.2 km/h (level 2)  • 3 minutes on the treadmill at speed 7.8 km/h (level 3)  • 3 minutes on the treadmill at speed 8.6 km/h (level 4)  • 4 minutes of cooling down on the treadmill 5.2 km/h (level 1)  Total time: 18 min |
| 4 | • 4 minutes of warm-up on the treadmill at speed 6.2 km/h (level 2)  • 4 minutes on the treadmill at speed 7.8 km/h (level 3)  • 4 minutes on the treadmill at speed 8.6 km/h (level 4)  • 3 minutes on the treadmill at speed 9.5 km/h (level 5)  • 4 minutes of cooling down on the treadmill 5.2 km/h (level 1)  Total time: 19 min |
| 5 | • 4 minutes of warm-up on the treadmill at speed 6.2 km/h (level 2)  • 4 minutes on the treadmill at speed 7.8 km/h (level 3)  • 3 minutes on the treadmill at speed 8.6 km/h (level 4)  • 3 minutes on the treadmill at speed 9.5 km/h (level 5)  • 2 minutes on the treadmill at speed 10.4 km/h (level 6)  • 4 minutes of cooling down on the treadmill 5.2 km/h (level 1)  Total time: 20 min |
| 5a | • 4 minutes of warm-up on the treadmill at speed 6.2 km/h (level 2)  • 4 minutes on the treadmill at speed 7.8 km/h (level 3)  • 3 minutes on the treadmill at speed 8.6 km/h (level 4)  • 3 minutes on the treadmill at speed 9.5 km/h (level 5)  • 1 minutes on the treadmill at speed 10.4 km/h (level 6)  • 1 minutes on the treadmill at speed 11.2 km/h (level 7)  • 4 minutes of cooling down on the treadmill 5.2 km/h (level 1)  Total time: 20 min |

Table S3. Protocol used to habituate the horses to handling and for the object tests. When a horse met the criteria for stage 5, it was considered ready for testing.

| **Stages** | **Description** |
| --- | --- |
| **Stage 1** | The horse can be taken out of the pen and accepts fitting of a halter. Positive reinforcement (feed and scratching on the neck) is used to motivate the horse to leave the pen and to accept the halter. |
| **Stage 2** | The horse walks forward, stops and turns in response to rope signals (negative reinforcement). |
| **Stage 3** | The horse enters the test arena area with the handler and eats from the feed container. |
| **Stage 4** | The horse accepts fitting of the heart rate equipment, incl. wetting of the skin and application of electrode gel. The horse eats from the feed container in the test arena while wearing the equipment. |
| **Stage 5** | The horse can be released by the entrance of the test arena and walks straight to the feed container and stays there eating for 120 s. |

Table S4: Ethogram of the recorded behaviors in the object tests.

| Behavior | Definition |
| --- | --- |
| Object focus | Vigilant with either neck raised over or below horizontal position, head and ears oriented towards the object. |
| Touch | Touching or manipulating the object (either umbrella, white plastic or buckets) |
| Sniff | Head within 20 cm of food container, neck horizontal or lower, clear exhalations from nostrils |
